# Network Pharmacology of Natural Products from Computational Prediction to Experimental Validation

**DOI:** 10.3390/biomedicines14102343

**Published:** 2026-10-15

**Authors:** Emmanuelle Usun Stephen, Khai-Lin Hew, Chze-Yin Tan, Yeun-Mun Choo

**Affiliations:** Department of Chemistry, University of Malaya, Kuala Lumpur 50603, Malaysia; emma.usun@gmail.com (E.U.S.); klhew4@gmail.com (K.-L.H.); chzeyin23@gmail.com (C.-Y.T.)

**Keywords:** network pharmacology, natural products, molecular docking, molecular dynamics, target prediction, polypharmacology

## Abstract

Natural products are important sources of bioactive compounds, but their complex chemical composition and multi-target effects make their mechanisms difficult to investigate using conventional approaches. Network pharmacology provides a systems-level framework for integrating chemical, biological, and disease-related information to investigate these complex interactions. This review describes the network pharmacology workflow, from active-compound screening, ADMET evaluation and target prediction to network construction and pathway enrichment, and the complementary roles of molecular docking and molecular dynamics. The workflow is organised around the strength of evidence, using a hierarchy that grades compound–target evidence, and the limits of validation and the items needed for reproducible reporting are specified. In addition, strategies for experimental validation, including biophysical and biochemical assays, cellular studies, omics approaches, pharmacokinetic evaluation, and in vivo models, are considered. Important limitations, including database bias, false-positive predictions, inappropriate network interpretation, limited consideration of in vivo exposure, and metabolite-related effects, are highlighted. Future developments involving multi-omics, microbiome data, single-cell and spatial analysis, and artificial intelligence may further improve the biological relevance and predictive value of network pharmacology. Overall, network pharmacology can serve as a useful bridge between natural product chemistry, computational analysis, and experimental pharmacology when supported by rigorous validation.

## 1. Introduction

Drug discovery has traditionally followed a “one-drug, one-target” approach, which is often insufficient for complex diseases such as cancer, neurodegenerative disorders, metabolic diseases, and antimicrobial resistance [1,2]. Single-target drugs may show limited efficacy, cause off-target toxicity, and promote drug resistance because they do not address the multiple pathways involved in disease progression [3,4,5,6,7]. These limitations have encouraged a shift toward polypharmacology, systems pharmacology, and network pharmacology, which consider multiple targets and biological pathways. The term “network pharmacology” was introduced by Andrew L. Hopkins in 2007 to describe the application of biological network concepts to drug action and drug discovery, and was subsequently developed as a broader drug-discovery paradigm [8,9]. In parallel, Shao Li and colleagues established early network-based approaches for traditional Chinese medicine (TCM), including the analysis of biomolecular networks associated with TCM syndromes and a framework for network-based investigation of Chinese herbal formulae [10,11].

Polypharmacology is the property of a compound acting on more than one target; it is what network pharmacology investigates, not a method. Systems pharmacology is broader, integrating molecular, cellular and physiological information, including pharmacokinetics/pharmacodynamics (PK/PD) and quantitative modelling, alongside network analysis [12,13,14,15,16]. Several related methods are distinct from network pharmacology but can supply inputs to it or test its outputs: chemoinformatics (computation of structural similarity, physicochemical properties and ADMET), AI/ML-based target prediction (learned compound–target models), molecular docking (prediction of ligand pose and score at a defined site), molecular dynamics (time-resolved simulation of a complex), and multi-omics pharmacology (measured molecular responses). Molecular docking and molecular dynamics therefore complement network pharmacology by helping to prioritize and provide computational support for network-derived target hypotheses; however, these approaches do not establish direct target interactions or experimental binding evidence, which require further experimental verification.

Medicinal plants are a long-standing source of structurally diverse bioactive compounds, such as withanolides, curcuminoids, bacosides, and triterpenoids, with documented anti-inflammatory, neuroprotective, hepatoprotective, and immunomodulatory activities [17]. Natural products (NP) are particularly suitable for a multi-target approach because their structural diversity, three-dimensional complexity, and variety of functional groups support interactions with multiple biological targets, and their structures have evolved to interact with biological systems [18,19,20,21,22]. NP can influence several pathways simultaneously and may be useful for treating complex diseases [23,24,25]. In a recent multi-evidence network pharmacology analysis of dietary flavonoids, the compounds were associated with a mean of 45.3 target proteins per compound, compared with 16.8 targets per FDA-approved drug in the reference dataset [26]; these associations are network-based predictions rather than direct evidence of target engagement.

Despite their potential, traditional NP discovery remains challenging. Complex mixtures of secondary metabolites make compound isolation, identification, and structural characterization difficult and costly, and low-abundance compounds and frequent rediscovery further reduce efficiency [27,28,29]. The multi-target nature of NP also makes their molecular targets and mechanisms difficult to determine [30,31]. Conventional in vitro assays and bioassay-guided fractionation remain important for initial characterization but may not reflect in vivo complexity, producing false-positive or false-negative findings [3,32,33,34].

Network pharmacology provides an integrated approach to address these limitations, complementing rather than replacing experimental methods. Modern techniques such as LC-MS/MS and metabolomics enable comprehensive chemical profiling of complex NP extracts [35,36,37,38]. Computational target prediction [39], integration with disease-related targets, and network and pathway analysis [40,41,42,43] can then generate hypotheses about hub proteins, pathways, multi-target mechanisms, and possible synergy [39,44,45]. However, predicted compound–target connectivity suggests potential multi-target activity and does not demonstrate polypharmacology, direct target engagement, or a therapeutic mechanism; these claims require experimental validation.

Existing reviews and guidance documents address network pharmacology broadly, set general standards for evaluating its methods [46], or discuss limitations of its data resources and analytical practice [47,48,49,50]. This review instead takes the strength of evidence as its organising principle and differs in three ways. First, it introduces an explicit hierarchy that grades compound–target evidence by the claim it supports (Section 2.4) and applies it throughout the workflow. Second, it defines the limits of validation: what computational predictions, docking, molecular dynamics, and each class of experiment can and cannot support, linking natural product-specific pitfalls such as under-represented scaffolds in prediction tools, exposure and metabolism, and species bias to that hierarchy. Third, it sets out the items that should be reported at each workflow stage to support reproducibility (Section 3), and follows representative studies from prediction to experimental outcome, including predictions that were not supported (Section 5.4).

This review draws on peer-reviewed literature identified through PubMed, CAS SciFinder, and Web of Science, searched from 2021 to 2026, with the most recent search conducted on 5 September 2026. Search terms combined natural product and phytochemical terminology with network pharmacology, target prediction, molecular docking, and molecular dynamics. Studies published within the last five years and journals ranked in the top two Journal Citation Reports quartiles were prioritized; earlier foundational references were retained where no comparable recent source existed. Applied studies illustrate methodological points rather than represent an exhaustive or systematic retrieval; quantitative claims are sourced to benchmark, guideline, or primary database and tool papers.

## 2. High-Throughput Analytics & Data Acquisition

### 2.1. Phytochemical Profiling & Dereplication

Phytochemical profiling and dereplication are important early steps in NP discovery. They enable rapid characterization of complex extracts, identification of known compounds, and prioritization of potentially novel metabolites for further study. These processes increasingly combine advanced analytical techniques with computational tools to improve the efficiency of NP discovery.

Modern metabolomics provides a comprehensive approach for investigating changes in the chemical composition of biological samples and relies on complementary analytical techniques, including LC-MS/MS, GC-MS, and NMR spectroscopy [51,52,53,54,55]. Each platform offers distinct advantages and is suited to different classes of compounds (Table 1).

LC-MS/MS is highly sensitive and versatile and can detect a broad range of polar and semi-polar compounds, including alkaloids, flavonoids, and terpenoids [56,57,58]. High-resolution instruments, such as UPLC-Q-TOF-MS, provide accurate mass measurements and high-resolution data that support the determination of molecular weights and isotopic patterns [59]. For example, UHPLC–Q-Exactive Orbitrap MS enabled the characterization of 170 compounds, including flavonoids, phenolic acids, and quinones, in Xiaoban Kangfu Capsules, a traditional Chinese medicine (TCM) formulation [60]. Similarly, UHPLC-Q-TOF-MS/MS enabled the profiling of flavonoids and saponins in *Passiflora* plants without the need for prior compound isolation [51]. GC-MS is particularly suitable for volatile and thermally stable compounds, including organic acids, fatty acids, and amino acids. It provides reproducible fragmentation patterns that can be compared with established spectral libraries [55]. For example, GC-MS analysis of *Platycladus orientalis* leaves identified 88 compounds and revealed age-dependent changes in compounds such as α-pinene and geraniol [61]. NMR spectroscopy provides complementary advantages for the direct and non-destructive analysis of complex mixtures. It can provide detailed structural information, including atom connectivity and stereochemical features [62,63,64,65]. Therefore, combining LC-MS/MS, GC-MS, and NMR can provide more comprehensive information on the chemical composition and characteristics of natural sources [66].

Molecular networking is a powerful tool for NP dereplication, particularly when dealing with complex extracts that are difficult to analyze using conventional isolation-based methods [67]. Platforms such as Global Natural Products Social Molecular Networking (GNPS) compare tandem MS/MS spectra based on their fragmentation patterns [38]. Structurally related metabolites are grouped into molecular families, allowing researchers to visualize and explore the chemical space of a complex extract [68].

By matching experimental MS/MS spectra with extensive community-curated spectral libraries, GNPS facilitates the rapid annotation and dereplication of known compounds and chemical families [38,69]. For example, GNPS analysis of *Buxus sinica* extracts enabled the visualization of the extract’s chemical space and the annotation of 21 distinct alkaloids [70]. Molecular networking can also highlight unannotated nodes that may represent previously undescribed compounds. This strategy contributed to the discovery of the novel cyclohexenone dimer aspergiflone A from *Aspergillus flavus* and the new cyclic lipodepsipeptide spectabilide A from *Trichothecium spectabile* [71,72]. Additional functions, such as MolNetEnhancer, further improve molecular networking by providing chemical-class annotations and helping researchers interpret the chemical diversity of complex extracts [73,74]. Nevertheless, GNPS and related approaches should be considered prioritization and dereplication tools rather than definitive methods for confirming compound novelty, structure, or biological activity. Their main value is to rapidly identify known compounds, organize chemical relationships, and direct researchers toward fractions or metabolites that warrant further investigation.

**Figure 1:**
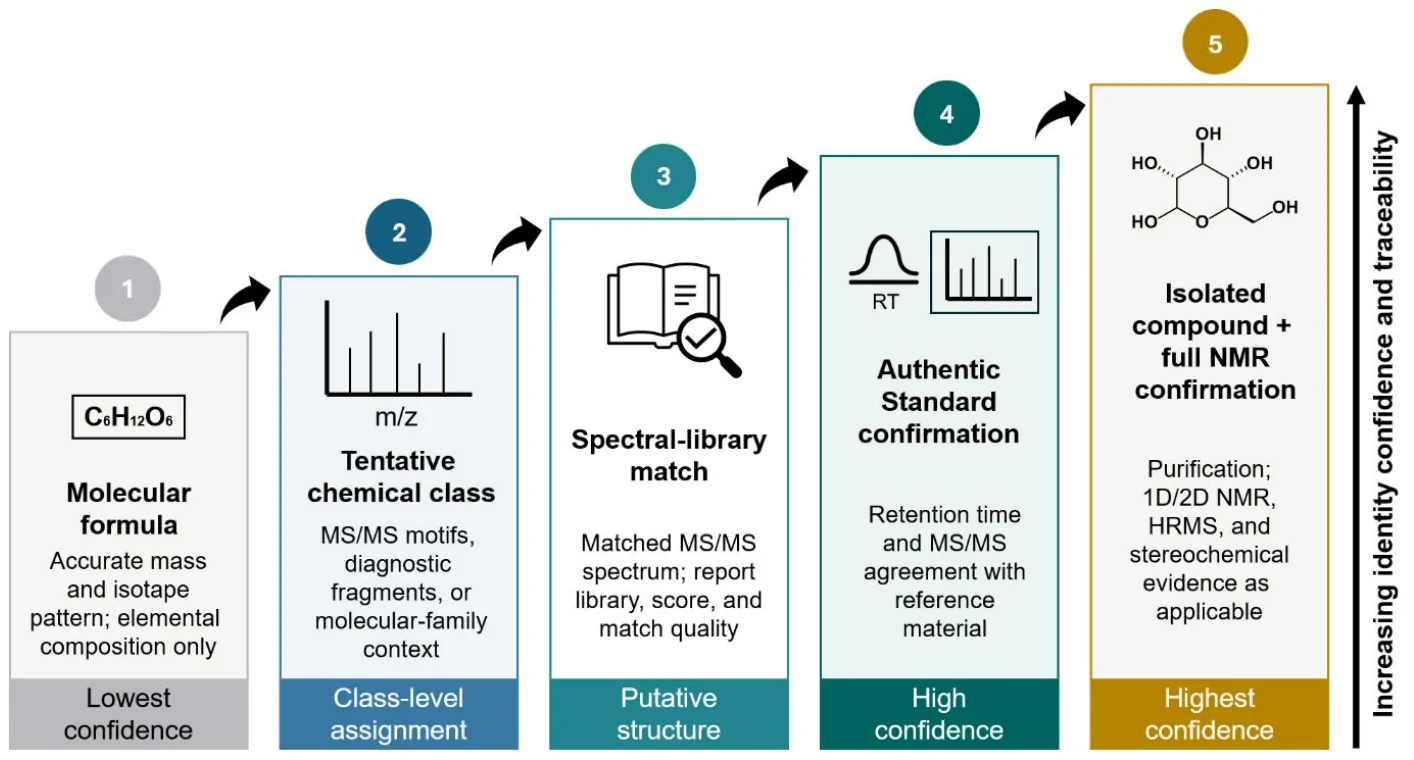
Workflow for natural product compound identification and structural characterization. Compound identification is reported at one of five increasing confidence levels, from molecular formula alone (Level 1) to a fully characterized, isolated compound confirmed by NMR (Level 5). The reported confidence level should reflect the actual analytical evidence available for a given compound, not be assumed uniform across a dataset.

Analytical detection alone cannot confirm the complete structure of a compound. The confidence of each compound identification should reflect the quality and extent of the supporting analytical evidence (Figure 1). This distinction is important for reliable compound identification and provides a strong basis for subsequent database integration, target prediction, and network pharmacology analyses.

### 2.2. Database Integration for Natural Products

**Figure 2:**
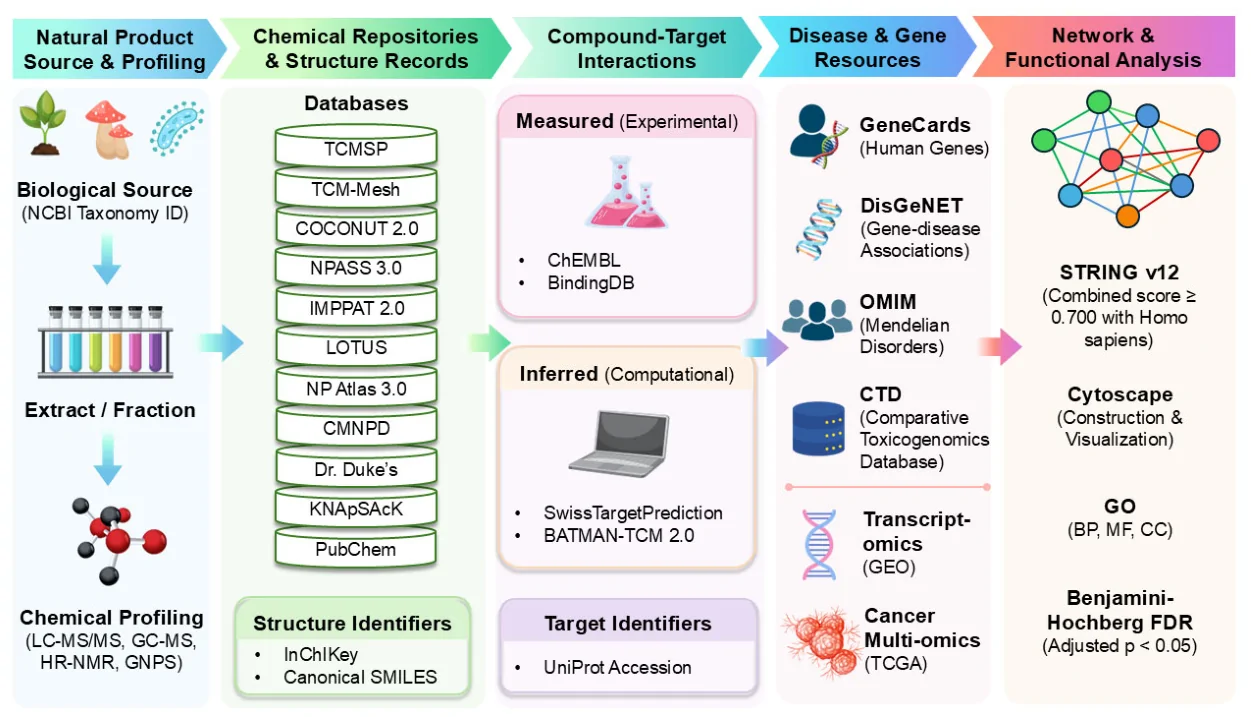
Workflow for the acquisition and integration of disease-associated target data in natural product network pharmacology. Natural product compounds identified and structurally characterized (Figure 1) are linked via stable structure identifiers to compound-target interaction data, classified as measured, inferred, or predicted, and standardized using UniProt accessions. This information is integrated with disease and gene resources to construct protein–protein interaction and functional networks for downstream analysis.

The compounds identified using the analytical platforms described in Section 2.1 must be converted into structured chemical and target information before network analysis can be performed. This subsection describes the databases that provide these data and the key retrieval parameters used. The resources covering the complete data pathway, from compound structures and targets to disease information and protein–protein interactions, are summarized in Table 2 and Figure 2.

For herbal medicines, the Traditional Chinese Medicine Systems Pharmacology database (TCMSP) is a widely used resource. It contains information on 499 pharmacopoeia herbs, 29,384 compounds, 3311 targets, and 837 disease associations, together with 12 ADME-related properties [75]. Commonly used screening conventions include oral bioavailability (OB) ≥ 30% and drug-likeness (DL) ≥ 0.18; however, these values should be applied as context-dependent prioritization criteria rather than universal exclusion thresholds. Notably, the original TCMSP study used an OB threshold of ≥40% in its liquorice example, indicating that the 30% threshold became a later convention rather than a value directly derived from the original study [75].

Broader natural product coverage is provided by several open databases. COCONUT 2.0 contains 695,133 unique natural product structures [77], while IMPPAT 2.0 provides 17,967 phytochemicals from 4010 Indian medicinal plants, with information on stereochemistry and FAIR data principles [79]. The LOTUS database contains more than 750,000 structure–organism records [80]. NPASS 3.0 further provides more than one million experimentally measured activity records and 208,415 records of compound concentrations in source organisms [78]. Microbial and marine natural products are covered by the Natural Products Atlas and CMNPD, respectively [81,82]. PubChem and ChEBI provide standardized chemical structures and chemical ontologies that can support data integration across these resources [85,86].

For plants outside these traditional systems, including many Southeast Asian, African, and Latin American species, ethnobotanical databases can provide useful starting information. Dr. Duke’s Phytochemical and Ethnobotanical Databases contain phytochemical composition and documented traditional uses [84]. The KNApSAcK database family links 20,741 species with 50,048 compounds and provides information on geographic distribution and traditional systems such as Kampo and Jamu [83]. However, these resources generally contain less target information than pharmacopoeia databases; therefore, compounds retrieved from them often require computational target prediction before network analysis.

### 2.3. Disease-Associated Gene and Target Databases

For complex diseases such as cancer, neurodegenerative disorders, and metabolic diseases, disease-associated gene and target databases provide essential information for network pharmacology analysis [39,50,95]. These resources collect complementary data on gene expression, disease–gene associations, genetic variation, and biological pathways, which can be integrated with natural product targets to identify potential therapeutic mechanisms (Table 2 and Figure 2).

The Gene Expression Omnibus (GEO) is an important source of transcriptomic data for identifying differentially expressed genes (DEGs) associated with specific disease conditions [96,97]. GEO datasets have been used to investigate molecular changes in diseases such as Parkinson’s disease, Alzheimer’s disease, hypertrophic cardiomyopathy, and vascular aging. They have also supported studies of natural products, for example the protective effects of piperine against retinal ischemic injury and the effects of *Paliurus ramosissimus* extract on metabolic pathways in colitis-associated colorectal cancer [98,99].

The Cancer Genome Atlas (TCGA) provides large-scale genomic, transcriptomic, and proteomic datasets across different cancer types [100,101]. These data can identify disease-associated transcriptional changes and help prioritise genes for further investigation when interpreted in the relevant tissue and cellular context. For example, TCGA RNA-seq data were used to identify altered MYC and CCND1 expression in prostate cancer in a study investigating the potential targets of Liuwei Dihuang Decoction (LWDHD) [102].

Several databases provide broader information on disease–gene and gene–phenotype relationships. Differentially expressed genes identified from GEO or TCGA should not automatically be interpreted as disease or therapeutic targets. Transcriptomic differences may reflect causal disease processes, compensatory responses, variation in cellular composition, immune or stromal infiltration, or downstream consequences of disease. Their interpretation should therefore consider tissue relevance, cell-type composition, disease stage, treatment status, direction and magnitude of expression change, sample heterogeneity, tumour purity where applicable, and technical factors such as platform differences and batch effects. Bulk transcriptomic findings should, where possible, be interpreted alongside cell-type-resolved, spatial, or complementary multi-omics data to distinguish disease-associated molecular changes from variation in tissue composition or microenvironmental context [103,104]. Candidate genes should be evaluated in independent cohorts and integrated with genetic, proteomic, functional, or experimentally curated disease evidence before being prioritised for network analysis or experimental validation [105].

Online Mendelian Inheritance in Man (OMIM), GeneCards, and DisGeNET are commonly used for this purpose. GeneCards integrates information from multiple sources, for example, it was used to identify 5678 disease-related genes in a study of crocin for spinocerebellar ataxia type 3 (SCA3) [106]. Similarly, the combined use of GeneCards, NCBI, and DisGeNET identified 7193 genes associated with heart failure in a study of Lu’an GuaPian tea [107]. The 5678- and 7193-gene sets reported above correspond to roughly a quarter to over a third of the protein-coding genome. Sets of this size make overlap with almost any candidate target list likely by chance alone, independent of the research bias toward well-studied genes discussed in Section 6.2. Filtering by the relevance or association score each database provides, and reporting the cutoff applied, narrows the set to genes with stronger disease-specific evidence and should be standard practice when using these resources for target prioritization. A formal test of whether an observed overlap exceeds chance expectation against an appropriately defined universe, or a comparison against a randomised set of unrelated compounds, is a specialised statistical undertaking beyond the scope of this workflow-level review. The Comparative Toxicogenomics Database (CTD) provides additional information on gene-disease, chemical-gene, and pathway relationships. For example, CTD identified 64 pathways and 15,667 genes associated with hyperuricemia in a study investigating the mechanism of isobavachin [108].

Overall, these databases provide complementary sources of disease-related information. GEO and TCGA are particularly useful for transcriptomic and multi-omics data, whereas OMIM, GeneCards, DisGeNET, and CTD provide broader disease–gene and pathway associations. Because their coverage and evidence types differ, combining multiple databases can provide a more comprehensive set of disease-associated targets for subsequent integration with natural product target data.

### 2.4. Target Mapping Resources and Retrieval Standardization

Compound–target relationships can be added to the network through three main evidence sources, which should be kept separate because they differ in reliability.

Measured interactions are supported by experimental evidence from primary studies and patents and are curated in databases such as ChEMBL and BindingDB under specific assay conditions [87,88]. However, these databases do not consistently provide information on natural product origin. In ChEMBL, the natural product label is based on a substructure filter and indicates that a compound is natural product-derived, rather than confirming its biological source [109].

Predicted interactions are obtained using ligand-similarity methods. SwissTargetPrediction compares compounds with a reference set of 376,342 bioactive compounds covering 3068 macromolecular targets. It identifies at least one correct human target among the top 15 predictions for more than 70% of tested compounds, and searches are commonly limited to *Homo sapiens* [89]. BATMAN-TCM 2.0 uses a similar approach for multi-component herbal medicines and distinguishes known interactions from predicted ones [90]. Measured and predicted evidence should be reported separately rather than merged into a single list, since the two carry different levels of confidence.

When multiple databases provide compound-target or disease-target information for the same entity, selection should be guided primarily by evidence tier and by how well a resource’s scope matches the biological question, such as its species coverage or disease focus, rather than by convenience or familiarity alone. Measured evidence from curated resources should generally be preferred over predicted associations when both are available for the same interaction. Discordant information between databases should not be silently resolved by combining sources or arbitrarily picking one; both records should be retained and reported, together with an indication of which evidence tier supports each, so that downstream analyses and readers can weigh them accordingly. Where a stable identifier such as InChIKey or a UniProt accession is available, it should be used to confirm that discordant entries genuinely refer to the same compound or target before concluding that a real disagreement exists, since apparent discordance can sometimes reflect an identifier mismatch rather than a true difference in evidence. The broader consequences of database fragmentation and inconsistent curation for prediction reliability are discussed further in Section 6.2.

Data should be linked using stable identifiers during retrieval rather than reconciled later. At the chemical and taxonomic levels, compounds can be linked using InChIKey and source organisms using NCBI Taxonomy identifiers, as applied in NPASS 3.0 [78]. Herb names are less reliable and should not be used as join keys. For example, dried tangerine peel may be listed in TCMSP as chenpi or *Citrus reticulata*, but in TCMID as Citri reticulatae pericarpium [50]. Since major databases are regularly updated, the database name, version, access date, and retrieval method should be recorded at each step to ensure that the compound–target dataset can be reproduced [46].

**Figure 3:**
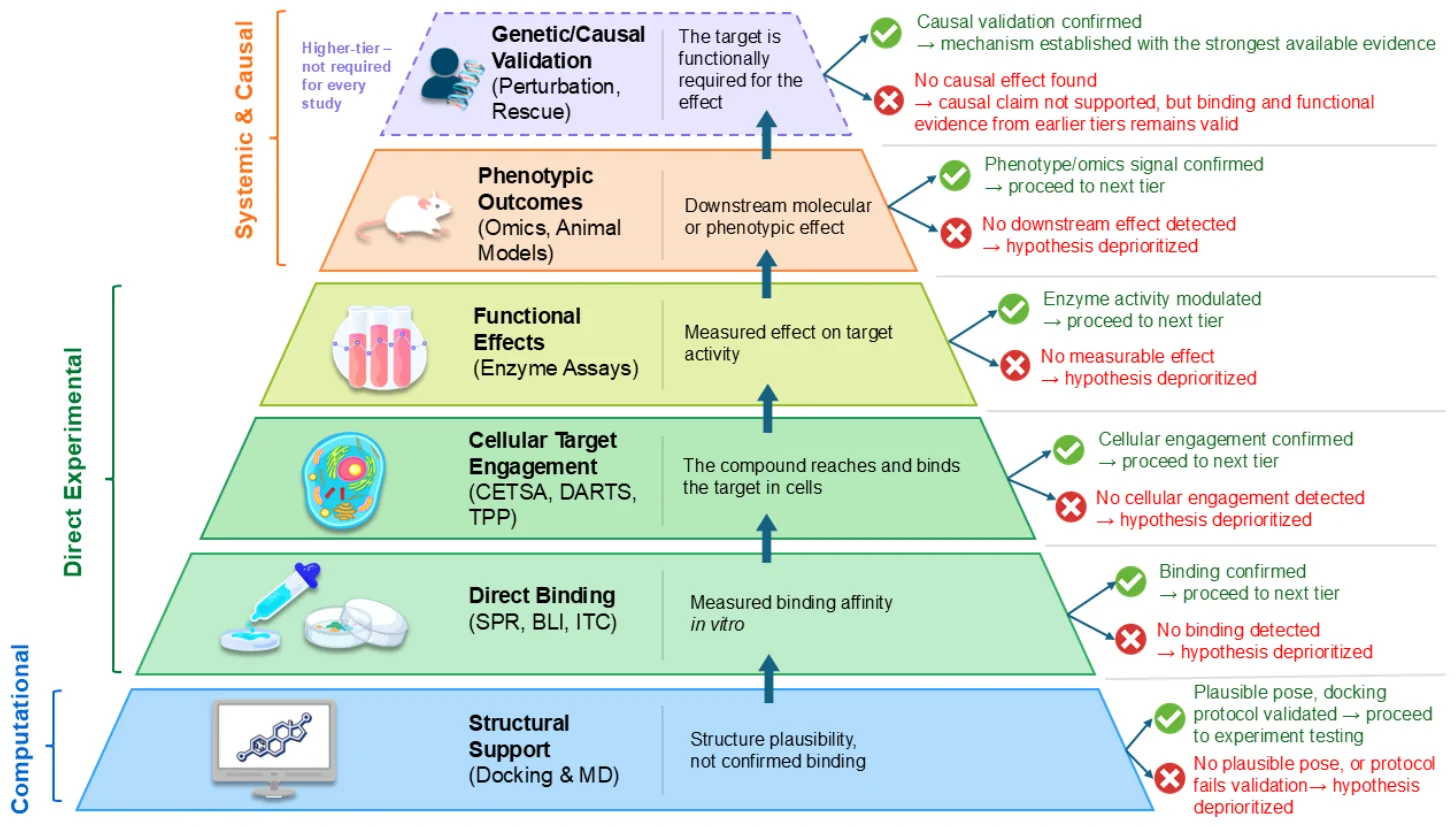
Integrated Computational Prediction and Experimental Validation Workflow. Compound-target evidence progresses from computational prediction (Structural Support: docking and MD) through increasingly direct experimental confirmation (Direct Binding; Cellular Target Engagement; Functional Effects) to systemic and causal evidence (Phenotypic Outcomes; Genetic/Causal Validation). At each transition, the criterion that advances a hypothesis to the next tier and the criterion under which it is instead deprioritized are shown explicitly. Genetic or causal validation provides the strongest support for a causal mechanism but is not required for every study; in its absence, binding and functional evidence from earlier tiers remains fully valid.

To make the strength of evidence explicit, we grade compound–target evidence in six tiers (Table 3). Structurally resolved interactions and experimentally measured binding or activity provide the strongest support for direct interaction, and cellular target engagement shows that the interaction occurs in cells. Evidence of downstream functional or phenotypic effects, such as genetic perturbation or in vivo efficacy at exposure-relevant doses, is graded separately in Section 5 and Figure 3.

## 3. Methodological Workflow for Network Pharmacology

Network pharmacology is a useful computational approach for investigating complex biological systems and disease mechanisms. It provides a systems-level view of how compounds interact with multiple molecular targets and biological pathways, which is particularly relevant to complex diseases where single-target approaches may be insufficient [16,27]. A typical network pharmacology workflow (Table 4) includes compound identification, active-compound screening, target prediction, disease-target identification, network construction and analysis, and functional and pathway enrichment. Molecular docking and molecular dynamics simulation are independent structure-based methods that complement this workflow and are listed separately in Table 4 [122,123].

### 3.1. Active Compound Screening and ADMET Evaluation

The initial step in network pharmacology is to identify and screen active compounds based on their absorption, distribution, metabolism, excretion, and toxicity (ADMET) properties. These filters help prioritize compounds with suitable drug-like and pharmacokinetic characteristics.

Lipinski’s Rule of Five is commonly used to assess drug-likeness based on molecular weight, hydrogen-bond donors and acceptors, and lipophilicity [124,125]. Generally, compounds with a molecular weight <500 Da, ≤5 hydrogen-bond donors, ≤10 acceptors, and MLogP ≤ 4.15 are considered to have favorable drug-like properties [126,127]. Commonly used OB and DL screening conventions are described in Section 2.2 and should be applied as context-dependent prioritization criteria alongside compound-specific pharmacokinetic and biological considerations [75,102,128].

For CNS-related studies, blood–brain barrier (BBB) penetration is an important consideration, while gastrointestinal absorption can also be assessed using tools such as SwissADME [129,130,131]. Caco-2 permeability and the apparent permeability coefficient (Papp) are commonly used to estimate intestinal absorption [125,132,133]. Toxicity prediction tools such as ADMETlab 3.0 and ProTox 3.0 can assess endpoints including AMES mutagenicity, hERG inhibition, acute oral toxicity, hepatotoxicity, and skin sensitization [126,131,134].

### 3.2. Target Prediction and Annotation

After active compounds are identified, their potential molecular targets can be predicted using computational methods. Similarity-based and machine learning approaches commonly assume that structurally similar compounds may interact with similar protein targets. For example, SwissTargetPrediction uses both 2D and 3D chemical similarity, while the Similarity Ensemble Approach (SEA) predicts targets based on chemical similarity [89,119,135]. PharmMapper uses 3D pharmacophore mapping to identify potential targets [136].

Other approaches include topological pharmacophore mapping, which provides a faster method for screening large compound libraries [137]. These ligand-based approaches are most informative when the query compound falls within the chemical space represented by the reference ligands; however, their performance may be limited for stereochemically complex, glycosylated, macrocyclic, or otherwise structurally unusual natural products that are under-represented in datasets dominated by synthetic, drug-like compounds [21,22,109]. Structure-based approaches, including reverse docking or inverse virtual screening, can complement similarity-based prediction by evaluating a query compound against panels of protein structures, although their performance depends on the quality of protein structures, binding-site definition, receptor flexibility, and scoring functions [120,138]. Consequently, ligand- and structure-based predictions should be considered complementary hypothesis-generating approaches (tier 4, Table 3) rather than interchangeable or confirmatory methods.

Machine- and deep learning drug–target interaction models represent a further, increasingly used alternative to similarity-based prediction, though they are typically trained on the same synthetic, drug-like chemical space described above, which may limit their reliability for structurally unusual natural products [139]. Agreement between two or more prediction tools is sometimes used to increase confidence in a predicted target, but this reasoning should be applied carefully. SwissTargetPrediction and the Similarity Ensemble Approach both rely on chemical similarity to reference ligand sets that overlap substantially, so agreement between them reflects shared input data as much as independent corroboration. Consensus across methods with genuinely different underlying evidence, such as a ligand-based prediction supported by a structure-based one, provides stronger support than agreement between two similarity-based tools alone. Reported performance varies across tools and test sets. In one benchmark using a newly compiled set of compounds, SwissTargetPrediction and the Similarity Ensemble Approach correctly identified a known target within the top ten predictions for roughly 60 to 64% of compounds, while a purpose-built consensus method reached about 66%, illustrating that no single tool is reliably superior across all cases.

Target coverage and prediction confidence also vary across platforms. For example, SwissTargetPrediction is principally designed for human macromolecular targets, whereas databases such as ChEMBL and BindingDB provide experimentally measured interactions under diverse assay conditions and may include data from multiple species [87,88,89]. Therefore, the selected target resource and species should match the biological question, particularly for anti-infective or other non-human applications. False-positive and false-negative predictions may arise from incomplete reference data, similarity bias, limited target coverage, promiscuous or assay-interfering structures, and sparse representation of the relevant biological species or natural product scaffold [49,109,140]. Where available, experimentally measured activity data should be prioritized over predicted associations, and the source, evidence class, score threshold, and rationale used to retain targets should be reported transparently.

Target names should also be standardized before network construction to ensure consistent data integration. UniProt identifiers are commonly used to standardize protein targets across different databases. Importantly, a high prediction probability or similarity score does not demonstrate biologically relevant target engagement, because the parent compound or an active metabolite must reach the relevant tissue and cellular compartment at a sufficient unbound concentration before it can exert a pharmacological effect [141,142].

### 3.3. Network Construction and Visualization

Network construction and visualization are important steps in network pharmacology because they provide a systems-level view of complex relationships among compounds, molecular targets, and diseases. Different types of networks can be constructed to represent these relationships and to explore the potential mechanisms of action of bioactive compounds.

Compound–target networks illustrate relationships between identified compounds and experimentally measured or computationally predicted protein targets. These networks are constructed by connecting identified compounds with their predicted targets and can demonstrate the multi-target or polypharmacological effects of compounds. Target-disease networks link potential molecular targets with genes associated with specific diseases, helping to identify possible therapeutic relationships. By integrating these networks, compound-target-disease networks can provide a broader view of the relationships among compounds, target proteins, and disease-related mechanisms. Such networks may reveal how multiple compounds act on interconnected targets and biological pathways.

Protein–protein interaction (PPI) networks are also important for understanding the functional relationships among target proteins. These networks can be constructed using databases such as STRING (Search Tool for the Retrieval of Interacting Genes/Proteins), which provides both known and predicted protein–protein interactions. STRING also allows users to filter interactions according to species, such as Homo sapiens, and confidence scores to improve the reliability of the selected interactions.

Cytoscape v3.10.5 is widely used to construct and visualize complex biological networks. It allows different node and edge attributes to be assigned, such as compound, target, or disease classifications and interaction confidence scores. Network layouts and visual properties can be adjusted to improve interpretation and highlight important nodes and interactions.

Network topology can then be analyzed to identify potentially important or highly connected nodes. Degree centrality measures the number of direct connections of a node and is commonly used to identify highly connected hub proteins. Betweenness centrality measures how frequently a node occurs on the shortest paths between other nodes, indicating its potential role as a key connector within the network. Closeness centrality measures the shortest-path distance between a node and other nodes, reflecting how efficiently it can interact with or influence the rest of the network. Centrality metrics should be calculated within the relevant connected component of a PPI network, often the largest connected component, because shortest-path–based metrics such as closeness and betweenness are not directly comparable across disconnected subnetworks [143,144]. Different centrality measures capture distinct topological properties of PPI networks, including local connectivity and shortest-path dependence [145]. Because cytoHubba algorithms quantify distinct topological properties and may yield different hub rankings [146], investigators should report agreement across complementary metrics or apply a pre-specified consensus rule rather than select hub proteins using a single centrality measure alone.

Standard local centrality metrics (Degree, Betweenness, Closeness) evaluate node importance based solely on immediate structural connectivity. However, unweighted degree centrality in raw interactomes heavily favors research-heavy ‘hub’ proteins (e.g., AKT1, TNF, TP53) due to extensive literature annotation bias in primary databases. To reduce this limitation, more advanced network analysis methods can be applied. Random Walk with Restart (RWR) simulates the movement of information through the network while allowing repeated returns to selected seed nodes, thereby considering the broader network structure rather than only direct connections. Network propagation and heat diffusion methods, such as HotNet2, model the spread of biological signals across the network and can identify groups of functionally related genes or proteins rather than focusing only on highly connected hubs. In addition, degree-preserving network randomization compares observed centrality scores with those obtained from 1000 randomly generated networks that maintain the original degree distribution. This approach allows calculation of a Z-score to determine whether a node has unusually high centrality and can help reduce the identification of false-positive network hubs.

### 3.4. Pathway and Functional Enrichment Analysis

Pathway and functional enrichment analysis provides biological context for the identified target genes and helps predict the molecular mechanisms underlying the effects of compounds. It is commonly performed using overlapping compound-disease targets and established biological databases to identify significantly associated biological functions and pathways.

Gene Ontology (GO) enrichment analysis classifies genes into three main categories: biological process (BP), molecular function (MF), and cellular component (CC) [24,147]. It can identify biological processes such as inflammatory response, apoptosis, oxidative stress, cell proliferation, and immune regulation; molecular functions such as protein kinase activity and receptor binding; and cellular components such as the plasma membrane, cytoplasm, nucleus, mitochondria, and extracellular region.

To reduce false-positive results from multiple testing, the Benjamini–Hochberg False Discovery Rate (FDR) correction is commonly applied, with an adjusted *p*-value < 0.05 generally considered statistically significant.

GO enrichment is often complemented by pathway resources such as KEGG and Reactome, and the choice of pathway database can change the enriched pathways, because equivalent pathways in different databases yield different results [148]. Enrichment tools such as DAVID [149], Metascape [150], clusterProfiler [151], and g:Profiler [152] draw on different, regularly updated annotation releases, and outdated annotations can change enrichment results [153]; the tool, its version, and the annotation release should therefore be reported. The background should also be stated and should contain the genes that could have appeared in the input list, which for predicted targets means the targets the prediction tool can return and not the whole genome, because a broader background can inflate significance [154]. Enrichment results indicate over-representation of pathway-related genes, not pathway activation or inhibition.

While network pharmacology readily identifies multi-target connectivity and shared biological pathways, predicted multi-target interactions represent polypharmacology or potential additive effects rather than proven synergy. To rigorously establish synergistic multi-component interactions, computational hypotheses must be evaluated against established mathematical reference models such as Loewe additivity, Chou-Talalay combination index, Bliss Independence, and other suitable models [29,155,156].

Although compound–target networks and pathway-enrichment analyses may identify constituents with potentially complementary biological actions, they cannot determine whether a combination produces additive, synergistic, or antagonistic effects. These interaction types require quantitative evaluation using experimentally measured combination-response data.

#### Synergy Quantification and Experimental Confirmation

Natural products, extracts, and multi-component formulations may contain constituents that act on different molecular targets or biological processes. However, the identification of multiple compounds, overlapping predicted targets, enriched pathways, or connected network modules does not itself establish pharmacological synergy. Such results may support a hypothesis of complementary or potentially additive activity, but synergy is demonstrated only when the observed effect of a combination exceeds the effect expected under a pre-specified non-interaction reference model. This distinction is especially important in network pharmacology, where compound–target connectivity and pathway overlap are hypothesis-generating rather than direct measurements of combination effects [29,128].

Several reference models can be used, but their assumptions differ and therefore their results should not be treated as interchangeable. Loewe additivity is based on dose equivalence: a combination is considered additive when one agent can be substituted by an effect-equivalent dose of the other. It is most suitable when compounds have similar or related mechanisms, or when their individual dose–response relationships can be meaningfully compared. The Chou–Talalay method similarly uses individual median-effect dose–response relationships to calculate a combination index (CI) at specified effect levels; conventionally, CI < 1, CI = 1, and CI > 1 indicate synergy, additivity, and antagonism, respectively. In contrast, Bliss independence assumes that the agents act independently on the measured phenotype. For fractional inhibition, the expected combination effect is *E_Bliss_*_,*expected*_ *= E_A_* + *E_B_* − *E_A_E_B_* = 1 − (1 − *E_A_*) (1 − *E_B_*). An observed combination effect greater than this expectation indicates Bliss synergy. If viability or survival fractions are used instead, the corresponding expected non-interaction response is *V_Bliss_*_,*expected*_ = *V_A_V_B_*, and synergy is indicated when the observed viability is lower than this expected value. The zero-interaction potency (ZIP) model estimates the expected response surface under the assumption that neither agent alters the potency of the other and quantifies the deviation of the observed response from this expectation. Because the null models capture different biological and pharmacological concepts, disagreement between Loewe, Bliss, Chou–Talalay, and ZIP results may be informative and should be reported rather than selectively presenting the most favourable score [157,158].

Appropriate synergy experiments require dose–response measurements for each monotherapy and suitable combination designs. A checkerboard experiment, comprising multiple concentrations of compound A crossed with multiple concentrations of compound B, enables response-surface analysis and identification of concentration-specific synergy or antagonism. A fixed-ratio serial-dilution design is particularly appropriate for Chou–Talalay analysis, provided that the selected ratio is justified, for example according to individual potency, clinically or physiologically relevant exposure, or the chemically characterized composition of an extract. For botanical extracts and multicomponent formulations, reproducible chemical characterization, reporting of major constituents where feasible, and batch-to-batch consistency are essential before an interaction can be attributed to a defined preparation or constituent combination [128].

Several practices can lead to overinterpretation. Synergy should not be inferred from shared network targets, common enriched pathways, molecular-docking scores, a superior combination response relative to untreated controls, or a single concentration pair without matched monotherapy comparisons. Conclusions can also be distorted by incomplete dose–response curves, use of concentrations outside plausible exposure ranges, inadequate technical or biological replication, endpoint-specific effects, and failure to specify the reference model. The full concentration matrix or fixed ratio, monotherapy controls, exposure duration, assay endpoint, replicate number, normalization method, reference model, and uncertainty estimates should therefore be reported transparently. SynergyFinder 3.0 enables analysis and visualization using Bliss, Loewe, highest-single-agent, and ZIP models and includes consensus interpretation across models; Combenefit provides interactive analysis and visualization based on Loewe, Bliss, and highest-single-agent reference models [158,159].

## 4. Complementary Structure-Based Computational Methods

Molecular docking and molecular dynamics (MD) are structure-based methods that are independent of network pharmacology but can complement it by assessing predicted compound-target interactions. Molecular docking evaluates potential binding modes and interactions, while MD assesses the stability and behavior of the predicted complexes over time. Together, these approaches help prioritize promising compounds for further experimental validation (tier 4, Table 3).

### 4.1. Molecular Docking

Network analysis can rank potential targets based on their network characteristics, but it does not confirm that a compound can bind to these targets. Molecular docking provides a structural approach to evaluate this possibility by predicting how a ligand may bind within a defined protein site and assigning it a docking score [120]. Thus, molecular docking is a useful tool for prioritizing compounds for experimental testing.

Protein structures are commonly obtained from the Protein Data Bank (PDB). When available, a high-resolution structure with a co-crystallised ligand is preferred because it provides a defined binding site and allows validation of the docking protocol. When no experimental structure is available, a homology model or an AlphaFold-predicted structure may be used, with caution, since such models are generated without a bound ligand and typically represent a single protein conformation. Ensemble docking, which samples multiple protein conformations, can partly address this limitation when the binding site is flexible [120]. Protein preparation generally involves selecting the relevant chain, removing crystallographic water molecules unless they contribute to binding, adding hydrogen atoms and partial charges, and assigning appropriate protonation states at physiological pH [138,160]. Docking methods differ in the flexibility allowed during the simulation. Rigid docking fixes both the protein and ligand, while semi-flexible docking allows ligand flexibility [161,162]. Flexible-receptor docking also allows movement of selected protein side chains and may be useful when binding causes structural changes [160]. This is particularly relevant for compounds with complex structures, many rotatable bonds, and stereocentres.

The search space should preferably cover the known binding site rather than the entire protein. Blind docking, which searches the whole protein, is commonly used in network pharmacology but can reduce prediction accuracy. Che and Zhang re-docked the 285 CASF-2016 complexes with a search box covering the whole protein, using AutoDock Vina 1.2.5 and QuickVina-W. Depending on the program and on exhaustiveness (8 or 64), 34–47% of the docking results reproduced the experimental pose within 2 Å, and the correlation between docking score and experimental affinity was r = 0.387 [138]. These figures are not directly comparable with CASF-2016’s own benchmark values for AutoDock Vina, a docking power close to 90% and a scoring power of about 0.6 [163], because CASF-2016 tests scoring on pre-generated poses at the correct binding site rather than on a whole-protein search. Blind-docking accuracy therefore depends on the benchmark used, the receptor, the search protocol, and the definition of success. Therefore, when a co-crystallised ligand is available, the docking box should be centred on its binding site, with the coordinates and dimensions clearly reported.

Docking protocols should be validated before screening test compounds. A common approach is to redock the co-crystallised ligand into the protein and compare the predicted pose with the experimental structure. An RMSD ≤ 2.0 Å is generally accepted as evidence of successful pose reproduction [163]. A known inhibitor or the co-crystallised ligand should also be included as a positive control.

A docking score is an empirical value, expressed in kcal/mol, that is intended to approximate experimental binding affinity [162]. It is not a measured or rigorously calculated binding free energy (ΔG) and should not be converted into a dissociation constant (K_d_) or an IC_50_ value. Throughout this review, “docking score” refers to the value returned by a docking program, and “binding affinity” is reserved for a measured quantity (K_d_, K_i_, IC_50_) or a value from a rigorous free-energy calculation. The Vina scoring function combines empirically weighted terms and is justified by its performance on test problems rather than by theory. Physics-derived terms in such functions must be weighed empirically to account for the difference between energies and free energies, and the receptor is treated as largely rigid [162]. Even when ligands are scored in their experimentally resolved crystal complexes, an idealised setting, most scoring functions evaluated in CASF-2016 correlated with measured affinities only at r = 0.21–0.63 [163]. In a critical review of docking-based screening for SARS-CoV-2 main protease inhibitors, docking scores from five programs correlated poorly with measured pIC_50_ values [120]. Docking generally predicts binding poses more reliably than binding affinity, and a favourable score alone does not support a claim of nanomolar or micromolar affinity. Both the docking score and the predicted interaction pattern should be reported, and scores should be compared only between ligands docked to the same target under the same protocol.

### 4.2. Molecular Dynamics Simulations

Molecular docking evaluates a static protein–ligand complex and provides only an approximate ranking of ligands rather than a quantitative estimate of binding affinity. Molecular dynamics overcomes some of these limitations by simulating the movement of the protein, ligand, and solvent over time using Newton’s equations of motion [164]. This allows the complex to relax and provides a more realistic assessment of its stability and binding behavior.

The docked complex is first solvated in explicit water, neutralized with counter-ions, and adjusted to physiological ionic strength. The system is then energy-minimized and equilibrated under constant volume and pressure before the production simulation [164]. Protein parameters are usually obtained from established force fields such as CHARMM36m or AMBER ff19SB [165,166]. Ligand parameterization is more challenging because different force fields and charge models can produce different binding-energy estimates [167].

There is no universal production length that guarantees adequate sampling of a system’s conformational space. The appropriate duration depends on the biological system under study, ligand flexibility, the conformational transitions of interest, the force field and solvent model used, and the specific properties being examined, so simulation duration alone should not be treated as a criterion for establishing complex stability. Trajectory length, sampling adequacy, and convergence are related but distinct considerations: a longer single trajectory does not by itself guarantee that the relevant conformational space has been adequately sampled, and because consecutive trajectory frames are correlated rather than statistically independent, simple averages computed across a single trajectory should be interpreted with caution. As a single simulation may not represent the full range of possible molecular motions, several independent replicas are increasingly recommended over one long trajectory. A large-scale replica study, using simulations of both a short peptide and a larger protein complex, found that even identically parameterised simulations can diverge substantially due to minor differences in initial conditions, and proposed a minimum of five to ten replicas as a practical rule of thumb, while noting that the number actually required depends on the question being asked and the reliability sought [168]. In practice, convergence can be assessed by discarding the initial equilibration period, comparing the properties of interest across independent replicas, and checking that these values stabilise rather than continuing to drift over time; consistency across replicas provides stronger evidence of a reliable result than a plateau observed in a single trajectory alone, since an apparent plateau can also reflect a kinetically trapped conformational state rather than true convergence.

Several trajectory measures are commonly used to evaluate complex stability [164]. Root mean square deviation (RMSD) measures structural changes over time, while root mean square fluctuation (RMSF) measures the flexibility of individual residues. Radius of gyration (R*_g_*) indicates changes in protein compactness, and hydrogen-bond occupancy measures how consistently specific interactions are maintained during the simulation. Stability should be assessed based on convergence and consistent plateaus rather than fixed numerical thresholds, because RMSD and other values depend on the size and flexibility of the system. Ligand RMSD is particularly important because a protein may remain stable even if the ligand moves away from the binding site.

Binding free energy can be estimated from molecular dynamics trajectories using Molecular Mechanics/Poisson–Boltzmann Surface Area (MM-PBSA) and Molecular Mechanics/Generalized Born Surface Area (MM-GBSA) methods. These approaches combine molecular mechanics energies with continuum solvation terms and are commonly used to compare the relative binding of different compounds [167,169]. Their results, however, are influenced by factors such as the choice of force field, sampling time, dielectric constants, and treatment of entropy. Benchmarking studies suggest that MM-PBSA may provide better estimates of absolute binding values, whereas MM-GBSA may perform better for ranking compounds in systems without metal ions [167]. Importantly, MM-PBSA and MM-GBSA results should not be directly compared with docking scores because the two approaches use different scoring and energy models.

A further limitation is that standard MM-PBSA/MM-GBSA calculations often omit the configurational entropy contribution because methods such as Normal Mode Analysis (NMA) and Quasi-Harmonic Analysis (QHA) are computationally demanding and can produce substantial statistical uncertainty. The calculation still includes a continuum solvation free-energy term even with entropy excluded, so the resulting value is not a measured enthalpy. It is more accurately described as an approximate end-point energy that leaves out the conformational entropy contribution, and it should not be treated as a true Gibbs free energy of binding or as an experimentally measurable quantity. This limitation is particularly relevant for flexible natural products, such as open-chain polyphenols and complex glycosides with many rotatable bonds, because their binding may involve substantial conformational entropy changes. To improve the interpretation of these calculations, the interaction entropy method can be applied to estimate conformational entropy changes directly from standard molecular dynamics trajectories without requiring additional extensive calculations. These estimates are also sensitive to the choice of force field, sampling protocol, and dielectric treatment. MM-PBSA/MM-GBSA results are therefore best used to rank ligands against each other under one consistent protocol, not as absolute values of binding free energy. Overall, molecular dynamics simulations together with appropriately interpreted MM-PBSA/MM-GBSA analyses can increase confidence in the structural stability and relative binding behaviour of predicted protein–ligand complexes, but their results should be considered comparative computational evidence rather than definitive experimental binding energies. Table 4 summarizes the network pharmacology workflow described in Section 2 and Section 3, together with the complementary structure-based computational methods described in Section 4, and the key items recommended for reporting at each stage.

## 5. Experimental Validation and Translation of In Silico Findings to In Vitro and In Vivo Results

Computational approaches, including network pharmacology, molecular docking, and molecular dynamics simulations, generate hypotheses about compound–target interactions and potential therapeutic mechanisms. However, these in silico predictions require experimental validation to confirm their biological relevance and potential therapeutic value. Validation can involve multiple levels, ranging from direct molecular binding assays to cellular, animal, phenotypic, and omics studies. Figure 3 summarizes the integrated workflow for validating predicted compound-target interactions through computational and experimental approaches.

### 5.1. In Vitro Validation of In Silico Predictions

In vitro validation tests predicted compound–target interactions at three levels of evidence: direct binding and target engagement (Section 5.1.1), functional and cellular effects (Section 5.1.2), and genetic dependence (Section 5.1.3). These levels correspond to the tiers in Table 3 and Figure 3 and support different claims, so a result at one level should not be presented as evidence for another. Earlier levels establish that a compound can bind or engage a target, whereas later levels test whether the target accounts for the observed biological effect.

#### 5.1.1. Direct Binding and Target Engagement

Direct compound-target interactions predicted in silico can be validated using several biophysical and biochemical assays. Surface Plasmon Resonance (SPR) and Biolayer Interferometry (BLI) are label-free methods that measure molecular binding in real time and provide kinetic parameters, including association rate (kon), dissociation rate (koff), and equilibrium dissociation constant (K_d_) [110,111,112,113,114]. Although both methods provide similar information, BLI may produce different K_d_ values from SPR because of factors such as analyte rebinding and mass transport effects [170]. These methods can provide in vitro evidence of direct binding to predicted targets.

Isothermal Titration Calorimetry (ITC) provides complementary information by directly measuring the heat released or absorbed during molecular binding. It determines binding affinity, binding stoichiometry, and thermodynamic parameters such as enthalpy (ΔH) and entropy (ΔS) [115]. Unlike SPR and BLI, ITC does not measure binding kinetics (k_on_ or k_off_). Cellular Thermal Shift Assay (CETSA) evaluates target engagement in intact cells by measuring changes in protein thermal stability after compound binding [171]. An increase in melting temperature can indicate ligand-induced protein stabilization, providing evidence that the compound engages its target under cellular conditions. Other label-free approaches can extend target validation beyond preselected protein. Drug affinity responsive target stability (DARTS) detects ligand-induced protection of proteins from protease digestion and can be combined with mass spectrometry to identify candidate targets without chemically modifying the compound [117,172]. Thermal proteome profiling (TPP) extends the thermal-stability principle of CETSA to proteome-wide analysis, identifying proteins whose thermal behaviour changes after compound treatment [118]. Chemical proteomics, including activity-based protein profiling (ABPP) and photoaffinity labelling, can likewise identify candidate binding proteins in complex biological samples through probe labelling, enrichment, and mass spectrometric analysis [173]. These approaches provide evidence of candidate target engagement or target identification, but hits should be confirmed by an orthogonal binding or functional assay before a direct mechanism is claimed.

#### 5.1.2. Functional and Cellular Validation

Enzyme inhibition or activation assays further assess the functional effects of compounds on enzyme activity. These assays commonly provide IC_50_ and K_i_ values and may help determine the mechanism of inhibition [40]. Western blot and RT-qPCR can be used to examine predicted hub proteins and pathway markers in relevant cellular models. These methods provide evidence of altered protein abundance or gene expression, respectively, but do not by themselves demonstrate direct target engagement. Their interpretation should include appropriate endogenous loading or reference-gene controls, vehicle controls, and, where possible, a positive control that produces the expected pathway response [174]. High-content screening can complement these assays by quantifying multiple image-based cellular phenotypes, including proliferation, apoptosis, migration, and morphology, after compound treatment [175,176].

Patient-derived organoids provide a more human-relevant model than conventional immortalised cell lines because they retain aspects of the architecture and heterogeneity of the tissue from which they are derived [177,178]. They may therefore be useful for evaluating whether network-derived hypotheses remain relevant in a disease-specific human model, although organoids do not fully reproduce systemic exposure, immune interactions, or all components of the native microenvironment. Across cellular models, dose–response rather than single-concentration experiments should be used, and test concentrations should be justified by measured or plausibly estimated in vivo exposure [179]. Positive controls and vehicle (solvent) negative controls, biological replication, and orthogonal assays that test the same claim by different principles reduce the risk of attributing an observed phenotype to an unsupported target interaction [180]. Recent natural product studies have combined chemical characterization, network pharmacology, molecular docking, and experimental validation to connect predicted targets and pathways with measurable anticancer effects. For example, Ci Gu Tang was investigated in pancreatic cancer using an integrated computational and experimental framework [181].

#### 5.1.3. Genetic Validation

Genetic perturbation can test whether a predicted target is necessary for the observed effect of a compound. siRNA/shRNA knockdown and CRISPR-mediated knockout can assess whether reduction or loss of a target attenuates the compound-associated phenotype [172,174], whereas overexpression can test whether increased target abundance alters the response. Rescue experiments, in which a version of the target that is resistant to the siRNA (for example, carrying silent mutations in the siRNA-binding region) is re-expressed, provide stronger evidence that the phenotype depends on the intended target and not on an off-target effect [182]. These approaches support causal interpretation but are not required for every exploratory study; direct binding, cellular target engagement, and functional evidence remain informative at their respective levels of claim.

### 5.2. In Vivo Models and Omics-Based Validations

Beyond direct binding and functional assays, omics approaches can help validate predicted pathways and molecular responses. Transcriptomics, including RNA-seq and scRNA-seq, measures changes in gene expression, while proteomics provides information on protein expression and post-translational modifications [183,184].

Functional enrichment analyses, such as Gene Ontology (GO) and Kyoto Encyclopedia of Genes and Genomes (KEGG) pathway analysis, are commonly used to interpret omics data. These analyses help link differentially expressed genes or proteins to specific biological processes and pathways. However, omics data can be affected by data quality, batch effects, and the complexity of analysing large datasets [185]. Transcriptomic data alone may also provide an incomplete view of biological responses because changes in gene expression do not always reflect post-translational modifications, metabolic changes, or epigenetic alterations that may contribute to disease progression or treatment resistance [103,104,105,186]. Therefore, integration of multiple omics approaches can provide a more comprehensive understanding of the molecular effects of candidate compounds.

Disease-relevant animal models are used to further evaluate efficacy, pharmacokinetics, and safety in vivo. Sprague–Dawley rats and C57BL/6J mice are commonly used in models of neurological, inflammatory, ischemic, and cancer-related diseases [128,144,187,188,189]. However, animal models may not fully reproduce the complexity of human disease, which can limit their predictive value for clinical translation [177].

### 5.3. Pharmacokinetics, Exposure, and Metabolite-Aware Validation

A predicted compound–target interaction is biologically plausible only when the parent compound or an active metabolite reaches the relevant tissue and cellular compartment at a sufficient unbound concentration [178,190]. Predicted affinity or a docking score should therefore not be interpreted independently of absorption, metabolism, plasma protein binding, tissue distribution, intracellular accumulation, target-site accessibility and elimination [191]. Total plasma concentration is not equivalent to the free concentration available to engage a target [190], and a cellular effect observed at a high nominal concentration may not be achievable in vivo [179]. Where possible, pharmacokinetic evaluation should measure or estimate parent-compound and metabolite concentrations in plasma and target tissue, determine the unbound fraction, and compare exposure with experimentally measured potency or affinity rather than with docking scores. Physiologically based pharmacokinetic modelling can support this comparison when adequate input data are available [3].

Compounds should be incorporated into the workflow rather than considered only after a parent-compound network has been constructed. LC–MS/MS-based metabolite identification and metabolomics can identify circulating metabolites [178,192], and in silico prediction of phase I, phase II and gut-microbial biotransformations can define candidate compounds before target prediction. Parent compounds and metabolites should then be represented as separate nodes, with metabolite-specific target predictions and experimentally measured activity data retained as distinct evidence classes [3,126]. This is especially important for botanical extracts and multi-component preparations, whose constituents may differ from those systemically available [193]. For isolated compounds, extracts and formulations alike, downstream cellular and in vivo experiments should use concentration ranges justified by measured or plausibly estimated exposure [179].

### 5.4. Worked Case Studies from Prediction to Evidence

The following three studies illustrate how computational workflows can reach different levels of evidence (Table 5). In each, computational methods (network pharmacology, docking or virtual screening and, where performed, molecular dynamics) generated target or compound hypotheses. In the first two studies, experiments confirmed phenotypic or pathway-level effects but did not demonstrate direct binding, cellular target engagement or genetic dependence for the prioritised targets, and some predictions were not supported. The third study reached direct biophysical binding by SPR but did not test cellular target engagement or genetic dependence. Together, the cases show that pathway-level confirmation, direct binding and functional relevance are distinct levels of evidence.

## 6. Limitations of Network Pharmacology Studies

### 6.1. Computational Network Analysis Without Experimental Validation

A major limitation in network pharmacology is the increasing number of studies that rely solely on computational analysis without subsequent experimental assessment. Although network construction using R v4.6.1, Cytoscape v3.10.5, and related tools can prioritize candidate targets, pathways, and compound–target associations, these outputs remain hypothesis-generating and cannot independently establish direct binding, cellular target engagement, causal mechanism, or therapeutic efficacy [46,195]. Accordingly, computational-only analyses should be interpreted as hypothesis-generating rather than confirmatory. The types of experimental evidence required to support direct binding, cellular target engagement, functional effects, and in vivo relevance are outlined in Section 5 and Figure 3.

### 6.2. Database Bias and False-Positive Predictions

Network pharmacology databases have important limitations, including fragmented data, differences in data curation, and variations in prediction methods [48]. Different databases use different inclusion criteria and data-processing methods, meaning that the choice of database can influence the predicted pharmacological results [47,49]. Data integration is also challenging because chemical structures may be represented using different identifiers, such as SMILES and InChIKeys [195]. Comparisons between databases often show limited overlap, leading to selection bias and inconsistent target information [49].

Disease-related databases such as GeneCards, OMIM, and DisGeNET may also contain more information on well-studied genes and pathways than on less-studied targets. This research bias can cause highly connected proteins, such as AKT1, TNF-α, COX-2, NFKB1, and RELA, and common pathways, including Nrf2/KEAP1/ARE, PI3K/AKT, MAPK, and NF-κB, to be repeatedly identified as key targets [39,196,197]. As a result, different natural products may appear to act through similar mechanisms, particularly those related to inflammation and apoptosis. To reduce the repeated prioritization of generic inflammatory hubs, investigators may complement default local centrality filters, such as those implemented in cytoHubba, with degree-preserving network randomization, Z-score-normalized centrality measures, or other context-appropriate network approaches.

Another limitation is the use of network connectivity to prioritize compounds. Common plant metabolites such as quercetin, kaempferol, and luteolin may appear as important compounds because they are associated with many predicted targets [198]. Some of these compounds are classified as Pan-Assay Interference Compounds (PAINS) and may produce non-specific effects through mechanisms such as membrane disruption, redox activity, metal chelation, or covalent binding [140]. Therefore, high target connectivity does not necessarily indicate specific or strong therapeutic activity. Experimental validation should carefully consider these potential false-positive effects to avoid overlooking more specific and biologically relevant compounds. To systematically handle these false-positive results, workflows should employ consensus scoring, requiring a compound-target interaction to be predicted by multiple independent algorithms before inclusion. Furthermore, stringent statistical corrections, such as false discovery rate adjustments, must be applied during pathway enrichment to eliminate spurious biological associations generated by large, noisy datasets.

### 6.3. In Vivo Exposure and Concentration Limitations

A major limitation of network pharmacology is the gap between the predicted activity of a compound and the concentration actually achieved in vivo. Some compounds may require high micromolar concentrations to interact with their predicted targets, while their levels in the blood after administration may be only in the sub-nanomolar range [142]. Although commonly used OB and DL screening conventions are described in Section 2.2, these criteria alone do not adequately reflect the dynamic nature of pharmacokinetics. For example, network studies of *Cordyceps* may predict direct target interactions for cordycepin without adequately considering its low systemic exposure and short in vivo half-life [141,199]. When a compound is poorly absorbed or rapidly eliminated, its concentration may not remain high enough to produce sustained direct effects on the predicted targets. The observed biological effects may instead involve indirect mechanisms, such as modulation of the gut microbiota or systemic immune responses, which are not well represented in conventional target-based networks. Therefore, rather than treating OB and DL values as absolute exclusion filters, workflows should incorporate exposure-aware screening. This includes the ADMET tools described in Section 3.1, complemented by physiologically based pharmacokinetic modeling and active metabolite profiling to account for in vivo exposure (Section 5.3). Approaches for pharmacokinetic and exposure-aware validation are described in Section 5.3. Such approaches can improve the biological relevance of network pharmacology predictions and reduce the risk of prioritising compounds that are unlikely to attain sufficient exposure at the relevant target site. Building upon this exposure-aware foundation, computational networks must be structurally adapted to reflect these physiological realities. For instance, indirect therapeutic mechanisms, such as gut microbiota modulation, can be explicitly incorporated as distinct network nodes. Furthermore, compound-target edges should be quantitatively weighted based on predicted systemic exposure levels to ensure centrality algorithms prioritize biologically accessible targets. To systematically resolve concentration limitations, simulated physiological concentration ceilings must be matched against measured affinities or potencies rather than docking scores, since the two are not directly comparable. If the molar concentration required to engage a specific target exceeds the compound’s achievable physiological limit, that interaction should be actively pruned from the network. Moreover, workflows can be adapted to evaluate polypharmacological synergy, mapping how multiple compounds acting simultaneously at sub-threshold concentrations might collectively modulate a pathway. Finally, to bridge the gap between computational prediction and physical validation, downstream cellular and in vivo assays must be strictly designed using these physiologically relevant concentration ranges rather than arbitrarily high doses.

### 6.4. Metabolite-Related Limitations

A common limitation in network pharmacology is the lack of consideration of human liver metabolism and gut microbiota biotransformation [141,142]. Many computational models assume that the parent compound identified in silico directly interacts with its predicted target in its original form [200]. However, many phytochemicals in botanical extracts have low oral bioavailability and may undergo extensive metabolic changes after administration [201]. Initial liver metabolism and biotransformation by the gut microbiota can convert the parent compound into metabolites that are more readily absorbed and reach the systemic circulation. These metabolites may have different chemical properties and target-binding profiles from the original compound used in the network analysis [202]. Therefore, integrating pharmacokinetic data and experimental validation is important to reduce false-positive predictions and to identify the compounds or metabolites that are responsible for the observed biological effects.

In addition, to computationally resolve this, active metabolite profiling must be integrated early in the pipeline. Workflows should construct enhanced networks that include both the parent natural products and their major predicted phase I, phase II, and gut microbiome biotransformation products. These complex parent-metabolite-target relationships can then be visualized using R packages such as ggalluvial or networkD3 to generate multi-layered Sankey flow diagrams that clearly delineate metabolic biotransformation pathways.

### 6.5. Species-Specific Database Constraints and Taxonomic Limitations

A critical but frequently overlooked constraint in network pharmacology is the overwhelming taxonomic bias toward *Homo sapiens* within primary computational target prediction tools and bioactivity repositories. Major ligand-based prediction platforms, such as SwissTargetPrediction, operate using reference datasets restricted almost exclusively to human macromolecular targets. Similarly, curated bioactivity databases like ChEMBL and BindingDB are heavily skewed toward human target assays, leaving target annotations for non-human species including pathogenic bacteria, viruses, fungi, parasites, and agricultural or veterinary organisms extremely sparse and fragmented.

This taxonomic imbalance fundamentally alters the predictive scope of network pharmacology in infectious disease research and antimicrobial natural product discovery. When evaluating antibacterial, antiviral, or antifungal plant extracts, standard computational workflows predominantly map compound interactions to human host proteins (e.g., host cell surface receptors, human inflammatory cytokines, or signaling kinases) rather than pathogen-encoded essential targets (e.g., bacterial cell wall synthases, viral proteases, or viral RNA polymerases). Consequently, target network predictions for anti-infective natural products inherently bias mechanistic hypotheses toward host-directed immunomodulation or host cell entry inhibition, while failing to map direct pathogen-targeted polypharmacological mechanisms. Crucially, the implications of this bias extend well beyond infectious diseases, severely impacting translational accuracy in fields such as veterinary pharmacology, agrochemistry, and ecotoxicology. At the molecular level, even when highly conserved orthologs exist, subtle variations in protein sequences can alter ligand binding site topologies, where a single amino acid substitution can abrogate binding affinity, rendering human-based target predictions unreliable for non-human orthologs. Furthermore, evolutionary divergences in baseline metabolism (e.g., varying cytochrome P450 profiles) and physiological signaling cascades dictate that a bioactivity network predicted using a human model may be mechanistically divergent or entirely absent in the target organism.

To navigate the human-centric bias of current network pharmacology databases, researchers must adopt rigorous, species-aware curation and validation strategies throughout their entire workflow. Initially, to prevent data fragmentation, target and compound extraction must rely strictly on standardized, immutable identifiers, such as SMILES strings, NCBI Taxonomy IDs, and UniProt accessions, rather than volatile regional or scientific nomenclature. As major predictive platforms predominantly output human targets, researchers cannot accept these annotations at face value for non-human studies; instead, they must actively cross-reference predicted human proteins using orthology tools (e.g., BLAST v2.17.0, OrthoDB v.12.2) to identify true homologous targets in the organism of interest. To further bypass the scarcity of non-human bioactivity data, these orthology mappings should be paired with independent, structure-based validation, utilizing molecular docking or dynamics simulations against non-human protein structures retrieved from the Protein Data Bank (PDB). Finally, during downstream pathway enrichment, users must critically filter the generated networks based on their specific biological context, such as explicitly segregating host-directed immune pathways from direct pathogen-targeted mechanisms, to prevent the inherent *Homo sapiens* bias of default biological databases from misdirecting the study’s overall mechanistic conclusions.

### 6.6. Methodological Constraints of AI-Driven Network Pharmacology

As network pharmacology increasingly adopts advanced computational models to improve target prediction, the application of Artificial Intelligence (AI) and Graph Neural Networks (GNNs) remains constrained by significant methodological limitations [203]. Crucially, reported performance metrics depend strongly on dataset composition, the specific prediction task, validation strategies, data leakage between training and test sets, and overall benchmark design [204,205]. Because these models rely on existing databases, they inherently absorb the same literature biases that skew traditional network centrality metrics [148,206]. Furthermore, these algorithms often demonstrate poor applicability to the complex, under-represented chemical scaffolds typical of natural products, as their training corpora are heavily dominated by synthetic pharmaceuticals [207]. Finally, the “black box” nature of deep learning introduces severe interpretability challenges, making it difficult for researchers to physically validate the algorithm’s underlying pharmacological rationale [208]. Therefore, before AI can effectively serve as a reliable engine for de novo polypharmacological design, future computational directions must prioritize the development of transparent, explainable AI architectures trained on structurally diverse, species-spanning datasets.

## 7. Future Perspectives and Emerging Directions

Network pharmacology is increasingly moving toward more integrated and precise approaches that combine artificial intelligence, microbiome information, patient-specific biological characteristics, and multi-omics data. These developments may improve the ability of network pharmacology to explain complex mechanisms and support the development of more effective therapeutic strategies. Single-cell multi-omics can further resolve cell-type-specific molecular programmes, tumour–microenvironment interactions, and intercellular heterogeneity that are masked in bulk-tissue datasets, thereby providing context for more biologically informed target and pathway prioritisation [209].

Artificial intelligence offers the potential to shift from static, rule-based network generation to dynamic, predictive pharmacology. Instead of relying on localized, static metrics like node degree, Graph Neural Networks (GNNs) leverage a sophisticated message-passing paradigm to learn continuous, high-dimensional vector representations [210,211]. The mathematical propagation within standard GNN layers allows computational models to capture highly complex, non-linear relationships between molecular structures and disease phenotypes. For instance, foundation models like TxGNN demonstrate robust capabilities in zero-shot drug repurposing, achieving an area under the precision-recall curve (AUPRC) of 0.913 for indications under conventional evaluations, and improving predictive accuracy by 19.0% over existing methods when identifying therapeutic candidates for diseases that lack approved treatments [212]. Building on these advancements, future workflows will utilize network target profiles to guide generative AI architectures, such as diffusion models and variational autoencoders [210]. By accurately predicting structure-activity relationships (SAR) directly from interaction networks, AI guides the rapid, iterative refinement of chemical structural backbones. This process enhances binding affinity while actively selecting against off-target toxicity, metabolic instability, and PAINS behavior, effectively shifting network pharmacology into a proactive engine for de novo polypharmacological design. This progress aligns with the framework of Network Target Theory, where AI operates across three synergistic tiers: Network Relationship Mining to identify relationships from large multi-omics datasets, Network Target Positioning to identify disease-related target networks, and Network Target Navigating to identify suitable therapeutic combinations [213]. However, as detailed in Section 6.6, realizing this potential requires overcoming foundational challenges in data quality, chemical diversity, and model interpretability before AI can function as a fully reliable engine for de novo drug design.

Integrating gut microbial genomic and metabolic algorithms into network pharmacology workflows represents a critical area for future network pharmacology research. The gut microbiome significantly influences both systemic immunity and the vital biotransformation of phytochemicals [214]. Integrating microbial genomic and metabolic data into network pharmacology may therefore help explain indirect therapeutic mechanisms. For example, some herbal formulations may reduce systemic inflammation by altering gut microbiota composition and lipid metabolism [214]. By fully accounting for microbial secondary metabolism and xenobiotic transformation, future computational models aim to provide a more holistic representation of drug action, spanning from initial ingestion to the downstream cellular response.

Beyond host-directed therapies, future network pharmacology workflows must expand into cross-kingdom and pathogen-targeted systems pharmacology to address infectious diseases and antimicrobial resistance. Current target prediction platforms remain heavily constrained by human-centric bias. Integrating pathogen-specific structural proteomes (including AlphaFold-predicted viral, bacterial, and fungal targets) with host–pathogen interactome networks will allow researchers to simultaneously map dual mechanisms: direct antimicrobial activity against microbial essential genes and indirect host immunomodulation. This multi-kingdom framework will be essential for unlocking the full therapeutic potential of anti-infective botanical extracts and marine natural products.

Network pharmacology may also support precision medicine by focusing on specific disease endotypes rather than treating diseases as uniform conditions. Osteoarthritis (OA), for example, can be classified into different endotypes, including bone-driven, synovitis-driven, and cartilage-driven forms, which involve different biological mechanisms [215,216]. The heterogeneity of these drivers explains why conventional approaches aimed at developing a single Disease-Modifying Osteoarthritis Drug (DMOAD) have faced repeated challenges [216]. AI-assisted network pharmacology may help analyse patient-specific multi-omics data to construct endotype-specific networks. When applied to the bone-driven endotype of OA, which is characterized by subchondral bone remodeling, osteoclast activity, and joint hardening, network pharmacology bypasses the cartilage entirely to focus on pathways regulating bone metabolism, such as the Wnt/β-catenin and RANK/RANKL/OPG networks. Conversely, for the synovitis-driven endotype, where chronic inflammation of the synovial membrane causes severe swelling and pain, the network model maps out a web of pro-inflammatory cytokines, targeting key hubs like TNF-alpha, IL-1beta, IL-6, and the NF-κB signaling pathway to identify effective multi-target anti-inflammatory compounds. Finally, for the cartilage-driven endotype, which involves the direct enzymatic destruction of the extracellular matrix, the analytical focus shifts toward matrix metalloproteinases (MMPs, particularly MMP-13) and ADAMTS enzymes, identifying compounds that can simultaneously inhibit these destructive elements while promoting cartilage repair. Ultimately, network pharmacology allows researchers to screen multi-target therapies, such as herbal formulas or drug cocktails, and precisely match them to the specific network disruptions of an individual patient’s endotype. For example, a bone-driven OA endotype may potentially be targeted using *Paederia scandens* and its bioactive compound asperuloside to specifically address subchondral bone changes.

Single-cell omics can provide detailed information on cell-specific responses and cell–cell communication. For example, single-cell studies have identified communication networks between ectopic stromal cells and M2-like macrophages in endometriosis [217,218]. Similarly, scRNA-seq has been used to investigate macrophage-to-myofibroblast transitions (MMT) in studies of Liangxue Siwu Decoction (LXSWD) for rosacea [219,220]. However, biological networks also depend on the spatial organization of cells within tissues. Spatial-omics approaches can complement single-cell analysis by linking molecular programmes to their local tissue context, including tumour–immune and stromal niches associated with therapeutic response or resistance [221]. Spatial transcriptomics can therefore complement single-cell data by mapping gene expression to specific locations within intact tissue [222]. This approach may help determine how a compound affects specific regions, such as tumour boundaries or inflammatory niches, and provide a more complete understanding of their biological effects.

## 8. Conclusions

Network pharmacology provides a valuable system-level framework for investigating the complex pharmacology of natural products. Its major strength is its ability to integrate chemical, molecular, and biological information to provide a broader understanding of multi-component and multi-target effects. High-throughput approaches, including LC-MS/MS, molecular networking, metabolomics, and NMR spectroscopy, can support the identification and dereplication of natural product constituents. When integrated with compound-target associations, disease-related genes, protein–protein interaction networks, and pathway analysis, these data can help prioritise promising compounds and generate mechanistic hypotheses that may be difficult to obtain using conventional single-target approaches.

An important advantage of network pharmacology is its ability to examine multiple targets within their broader biological context. Network pharmacology can connect multiple compounds, targets, pathways, and disease processes within an interconnected network. This broader perspective is particularly useful for natural products, which often contain multiple constituents that act on multiple targets through polypharmacological mechanisms, with complementary or overlapping effects across interconnected biological pathways. It can therefore help explain how multi-target effects may contribute to therapeutic responses, identify potential target combinations, and distinguish biological connectivity from simple target multiplicity. Network pharmacology can identify constituent–target–pathway relationships that generate hypotheses of complementary or potentially synergistic activity; however, pharmacological synergy requires dedicated combination experiments and quantitative comparison with a pre-specified reference model.

The integration of molecular docking and molecular dynamics can provide additional structural evidence for predicted compound-target interactions. Similarly, combining network pharmacology with metabolomics, pharmacokinetics, transcriptomics, proteomics, single-cell and spatial omics may provide a more complete understanding of how natural products influence biological systems. These approaches can help identify not only potential targets but also relevant metabolites, cell populations, tissues, pathways, and disease endotypes. Artificial intelligence and graph-based methods may further improve the prediction of complex compound-target relationships and support the optimisation of natural product-inspired multi-target compounds.

Despite these advantages, network pharmacology should not replace experimental research. The reliability of network pharmacology results depends on the quality, provenance, and context of the underlying data, including chemical annotation, database information, target evidence, and disease relevance. Important limitations include database and literature bias, repeated identification of highly connected compounds and hub proteins, fixed ADME thresholds, insufficient consideration of achievable in vivo exposure, and limited incorporation of hepatic and gut microbiota-derived metabolites.

Future studies should adopt context-specific, reproducible workflows that grade compound–target evidence by the claim it supports, state what each computational and experimental step can and cannot establish, and report the items needed to reproduce each stage. Predicted targets and mechanisms should be evaluated using complementary biophysical, biochemical, cellular, omics, pharmacokinetic, and in vivo approaches. Overall, network pharmacology is most valuable when used as a bridge between natural product chemistry, computational biology, and experimental pharmacology. By integrating chemical profiling with biological networks and rigorous experimental validation, it can help transform the complexity of natural products into credible, reproducible, and testable multi-target therapeutic opportunities.

## Figures and Tables

**Table 1:** Comparative strengths and limitations of analytical approaches used for NPs profiling and dereplication in natural product discovery.

Methods	Chemical Coverage	Sensitivity	Structural Certainty	Principal Limitations
LC-HRMS/MS	Broad; polar to semi-polar compounds	Very high	Moderate without standards/NMR	Ionisation bias; isomers; MS/MS annotation ambiguity
GC-MS	Volatile and derivatisable compounds	High	Moderate-high with library match and retention index	Limited for non-volatile/thermolabile compounds; derivatisation may be needed
NMR Spectroscopy	Broad but concentration-dependent	Low-moderate	High connectivity and stereochemistry support	Lower sensitivity; overlap in complex mixtures; larger sample requirement
Molecular networking (GNPS/FBMN)	MS/MS-detectable chemical families	Depends on upstream LC-MS/MS	Low-moderate unless matched to standards	Clusters spectral similarity; not proof of identity, novelty, abundance or bioactivity

**Table 2:** Important databases for natural product network pharmacology. All resources were accessed on 28 August 2026.

Resource	Core Category	Primary Functional Role and Architecture	Selection Parameters and Commonly Used Filters	Coverage Constraint
TCMSP [75]	Phytochemical & ADME (curated + predicted)	Chinese herbal chemistry, ADME parameters, target links	Oral Bioavailability (OB) ≥ 30%; Drug-Likeness (DL) ≥ 0.18	Redundant entries on herb-name query; Chinese herbal medicines only (499 pharmacopoeial herbs); last major update 2014
TCM-Mesh [76]	Herb preparation (curated)	Herb-compound-target-disease relations with toxicity annotation	Compound and herb name query	Fewer ingredients per herb than TCMSP or TCMID; TCM herbs and formulae only; no confirmed update since 2017
COCONUT 2.0 [77]	Natural repositories (curated structural aggregation)	Aggregated open structural annotations, community-curated	SMILES/InChIKey extraction; taxonomic filtering	Organism annotation provided for a subset of entries; multi-species, aggregated from over 63 sources; current as of 2025
NPASS 3.0 [78]	Quantitative bioactivity (measured)	Activity, ADME-Tox and source-organism composition records	Activity class; organism source; assay level	47,418 of 208,415 NPs (22.75%) carry quantitative activity annotation; multi-species (48,940 source organisms); current as of 2026
IMPPAT 2.0/LOTUS [79,80]	Phytochemical atlas/structure-organism (curated)	Part-resolved Indian plant chemistry; referenced structure-organism pairs	Stereo-aware library; referenced pair validation	IMPPAT restricted to Indian flora (last update 2023); LOTUS holds occurrence data only, multi-species (last update 2022)
NP Atlas 3.0/CMNPD v1 [81,82]	Microbial & marine NP (curated)	Microbially derived and marine natural product structures	Taxonomic and structural query	Source-restricted by design to microbial and marine organisms; NP Atlas current as of 2024/2025, CMNPD last update 2021
Dr. Duke’s/KNApSAcK [83,84]	Ethnobotanical & regional (curated)	Ethnobotanical use records, quantitative phytochemical content, and species-compound relationships for non-pharmacopoeial flora	Plant name, chemical, biological activity or ethnobotanical use query	Occurrence and use data with limited target annotation; Dr. Duke’s content derives from the 1992–2016 compilation, not actively updated since; KNApSAcK last update 2012
PubChem/ChEBI [85,86]	Structural databases (curated)	Canonical structures, 3D conformers, chemical ontology	Canonical SMILES; 3D SDF; InChIKey	Deposit-dependent quality; no natural product flag; not species-restricted; continuously updated
ChEMBL/BindingDB [87,88]	Measured bioactivity (measured)	Curated quantitative affinities from primary literature and patents	Assay type; activity cutoff; target confidence	NP provenance not curated; ChEMBL NP flag is substructure-derived; multi-species assay data, not restricted; ChEMBL current as of 2024, BindingDB as of 2025
SwissTarget Prediction [89]	Target prediction (predicted)	Reverse ligand-based mapping via 2D/3D similarity	Probability score cutoff; restricted to *Homo sapiens*	~30% of queries return no correct target within top 15; restricted almost exclusively to *Homo sapiens*; no confirmed major update since 2019
BATMAN-TCM 2.0 [90]	Target prediction (predicted + known, distinguished)	Known and predicted interactions for multi-ingredient formulae	Adjusted *p*-value cutoff; target score threshold	Prediction confidence varies with training coverage; not species-restricted; current as of 2024
GeneCards v4/DisGeNET v7.0 [91,92]	Disease genomics (mixed: curated, inferred, text-mined)	Integrated human genetic, transcriptomic and disease mapping	DisGeNET score ≥ 0.1 or top-quartile specificity	Pools curated, inferred and text-mined associations under one score; human-focused
OMIM/CTD [93,94]	Phenotype & toxicogenomics (curated)	Mendelian gene–phenotype relationships; curated chemical–gene–disease interactions	Direct expert-curated disease-gene matching	OMIM restricted to Mendelian phenotypes, human only; CTD cross-species, requires taxon filtering
STRING v12 [43]	Protein interactome (mixed: experimental, curated, text-mining)	Physical and functional protein–protein associations	Combined score ≥ 0.700	Text-mining channel contributes at all score thresholds; multi-species, filterable (e.g., *Homo sapiens*)

**Table 3:** Evidence hierarchy for compound–target interactions in natural product network pharmacology.

Tier	Evidence	Typical Sources or Methods	Supports	Does Not Support
1	Structurally resolved interaction	Co-crystal, cryo-EM, NMR	Binding pose at a defined site	Cellular activity or exposure
2	Experimentally measured interaction	SPR, BLI, ITC, enzyme assays, curated ChEMBL or BindingDB values [110,111,112,113,114,115]	Direct binding or activity in vitro	Engagement in cells
3	Cellular target engagement	CETSA [116], DARTS [117], TPP [118]	The compound reaches and binds the target in cells	A causal role in the phenotype
4	Computationally predicted interaction	Similarity tools [89,119], ML models, reverse docking, docking and MD [120,121]	A testable hypothesis	Binding or activity
5	Indirect network association	Shared pathway, PPI neighbour, enrichment overlap [43]	Biological plausibility	A direct interaction
6	Literature or text-mined association	Text-mined records, DisGeNET-type inferred links [43,92]	A lead for curation	Any experimental support

**Table 4:** Overview of the Network Pharmacology Workflow and Complementary Structure-Based Computational Methods.

Workflow	Key Methods	Purpose	Key Reporting Items and Limitations
Active compound screening and ADMET evaluation	Assess drug-likeness, oral bioavailability, absorption, and toxicity.	Select compounds with suitable drug-like, pharmacokinetic, and safety properties	Screening criteria and ADMET tool/version; thresholds alone do not capture in vivo exposure (Section 6.3)
Disease-target retrieval	Query disease-gene and multi-omics resources (GeneCards, DisGeNET, OMIM, CTD, GEO, TCGA); link via NCBI Taxonomy ID and UniProt accession	Identify genes and pathways associated with the disease of interest	Database version, relevance/association score cutoff applied; large disease-gene sets approach genome-scale coverage and should be filtered rather than used unfiltered (Section 2.3)
Target prediction and annotation	Use chemical similarity, machine learning, and pharmacophore-based methods. Standardize targets using UniProt.	Identify and organize potential molecular targets.	Tool, version, score cutoff, and UniProt mapping; limited by training-data and species bias (Section 6.2 and Section 6.5).
Network construction and visualization	Build compound-target, target-disease, compound-target-disease, and PPI networks. Use STRING v12 and Cytoscape v3.10.5. Analyze key nodes using network centrality.	Visualize relationships and identify important targets or hub proteins	PPI resource, confidence threshold, and centrality method; degree centrality favours well-studied hubs (Section 6.2)
Pathway and functional enrichment analysis	Perform GO and pathway enrichment analysis and apply statistical correction.	Identify important biological functions and pathways associated with the targets	Enrichment database and correction method; results are hypothesis-generating, not confirmatory
Synergy quantification and experimental confirmation	Monotherapy dose–response curves; checkerboard or fixed-ratio combination designs; Loewe, Bliss, Chou–Talalay CI, and ZIP models; SynergyFinder; Combenefit.	Experimentally distinguish synergy, additivity, and antagonism from network-inferred multi-target activity	Reference model, monotherapy controls, and replicates; network overlap alone does not establish synergy
Molecular docking	Predict ligand binding pose and assign a docking score against a defined protein site.	Assess structural plausibility and prioritize compounds for experimental testing	Program, box settings, and redocking RMSD; scores rank poses within one protocol, not absolute affinity
Molecular dynamics simulation	Simulate protein-ligand-solvent motion over time; assess stability with MM-PBSA/MM-GBSA.	Provide comparative computational evidence of complex stability and relative binding behaviour	Force field, replicas, and convergence check; a single trajectory cannot establish convergence

**Table 5:** Representative Case Studies Tracing Network Pharmacology Predictions to Experimental Outcomes.

Study/Preparation	Computational Workflow & Prediction	Experimental Validation Performed	Evidence Tier Reached	Confirmed Findings vs. Prediction Failures/Gaps
Liuwei Dihuang Decoction [102]	TCMSP & docking predicted 6 core targets (TP53, AKT1, MYC, TNF, CCND1, MAPK1) in prostate cancer.	In vitro PC3 cell assays (CCK-8, wound healing); qPCR and Western blot	Tier 4 (docking). Functional and pathway-level effects in cells; no tier 1 to 3 evidence.	Confirmed: Suppressed cell migration/proliferation; downregulated PI3K/AKT. Not supported: No significant effect on MAPK1, or MYC proteins. Direct physical binding remains unverified.
Anti-JEV Botanical Library [40]	SwissADME & CDOCKER predicted 6 BBB-permeable compounds targeting viral enzymes and host PTGS2/NF-*κ*B	BV2 & Neuro2a cells (qPCR, Western blot, plaque assays, *IC*_50_)	Tier 4 (predicted links). Phenotypic and functional effects in cells; no target-level evidence.	Confirmed: 3 compounds inhibited PTGS2/NF-*κ*B and JEV replication. Not supported: 3 compounds did not reduce inflammatory markers; indigo did not reduce viral replication when added after infection. Target engagement unverified
4′-O-Methylochnaflavone [194]	Transcriptomics, virtual screening, and MD simulations predicted HSP90AB1 as a target in HCC.	Surface Plasmon Resonance (SPR); Hep-3B cell assays (CCK-8, Western blot).	Tier 2 (SPR direct binding). Cellular modulation of HSP90AB1/HIF-1α; no tier 3.	Confirmed: SPR proved direct physical binding (*K_D_* = 2.97 μM), downregulated hypoxia-induced HSP90AB1/HIF-1α Gaps: Lacks cellular target engagement (tier 3), genetic dependence validation and in vivo pharmacokinetics.

## Data Availability

No new data were created or analyzed in this study. Data sharing is not applicable to this article.
